# COVID-19 epidemic lockdown-induced remarkable decrease in dairy products consumption of Iran population: does it really matter? National Food and Nutrition Surveillance

**DOI:** 10.1186/s40795-022-00612-w

**Published:** 2022-10-27

**Authors:** Bahareh Nikooyeh, Samira Rabiei, Maryam Amini, Delaram Ghodsi, Hamid Rasekhi, Azam Doustmohammadian, Zahra Abdollahi, Mina Minaie, Farzaneh Sadeghi, Tirang R. Neyestani

**Affiliations:** 1 Department of Nutrition Research, National Nutrition and Food Technology Research Institute and Faculty of Nutrition Sciences and Food Technology, Shahid Beheshti University of Medical Sciences, Tehran, Iran; 2 Gastrointestinal and Liver Diseases Research Center (GILDRC), Iran University of Medical Sciences, Tehran, Iran; 3 Community Nutrition Office, Deputy of Health, Iran Ministry of Health and Medical Education, Tehran, Iran

**Keywords:** Covid-19, Dietary habits, Dairy products, Consumption frequency, Survey

## Abstract

**Background:**

The pandemic of the newly emerged coronavirus infection and its related disease, Covid-19, has influenced various aspects of human life including dietary habits. This study aimed to examine changes in dairy products consumption during Covid-19 lockdown period in a huge sample of Iranian households.

**Methods:**

A cross-sectional descriptive-analytical study using a web-based electronic self-administered questionnaire designed to detect any changes in the consumption frequency of dairy products in the Iranian households during Covid-19 lockdown.

**Results:**

A total of 21,290 households were enrolled. During Covid-19 epidemic lockdown, about 29%, 26% and 7% of the households had decreased their consumption frequency of milk, yogurt and cheese, respectively. The female-headed households were 21% more likely to decrease their consumption of milk, compared with male-headed households (OR = 1.21, 95% CI: 1.05–1.4). The households residing in food insecure provinces were 29%, 20% and 45% more likely to decrease their consumption of milk, yogurt and cheese as compared with those living in the food secure provinces. About 37%, 25.3%, 19.4% of those households who reported a decrease in consumption of dairy products had fully omitted them.

**Conclusion:**

We found considerable decrement of dairy products consumption, especially milk and yogurt, in a high proportion of the studied households. Inadequate intake and, in some households, omission of dairy products can potentially bring about serious health outcomes with heavier economic burden. Further studies to track these changes over time and to evaluate their health consequences are warranted.

## Background

Emergence of the new coronavirus infection in Wuhan, China, in the late 2019 was followed by its rapid spread globally. The related disease, severe acute respiratory syndrome (SARS-Covid-19) brought about millions of morbidities and mortalities all over the world [1]. Consequently, social distancing was advised by all scientific bodies and meanwhile many countries had to lock down all activities with the hope to reduce viral transmission. This situation inevitably affected many, if not all, aspects of human life including socioeconomic, educational and eating habits, just to name a few [2].

Many businesses especially retails were severely damaged with consequent remarkable decrease in the households’ incomes. To this, fear of viral transmission either through respiratory droplets during shopping or either via food items purchased from groceries caused a significant change in eating habits. Obviously, these changes can be different from one community to another [3]. Some of these changes might be toward healthy eating including adherence to Mediterranean diet, eating more fruits and vegetables [4, 5] and decrease in fast foods and alcohol consumption [5, 6]. On the other hand, in some communities unhealthy dietary changes such as increased consumption of high calorie snacks and alcohol have been reported [7]. Among the changes of dietary habits, dairy consumption is of special importance due to its direct relevance with such health outcomes as bone density and metabolic health [8, 9]. Though initially it was thought that these changes would be transient with minimal health effects, persistence of coronavirus infection throughout the world proved it was wrong. This study was, therefore, undertaken to examine changes in dairy products consumption during Covid-19 lockdown period in a sizable sample of Iranian households.

## Methods

### Study design and population

We conducted a cross-sectional study using a web-based electronic self-administered questionnaire specifically designed to detect any changes in the dietary pattern of the Iranian household following coronavirus epidemic, as comprehensively described elsewhere [10]. A panel of nutrition experts (comprising of seven internal and three external specialists) confirmed the content validity. Then, the questionnaire was uploaded on a web link. The vice-chancellors in health affairs and the Community Nutrition Offices of the medical universities of all provinces were informed of the objectives of the project through an official letter sent from the Community Nutrition Office, Deputy of Health, Iran Ministry of Health, Treatment and Medical Education (MOH). In that letter, nutrition officers and health workers were requested to notice the community under their service coverage and encourage them to participate in the project. To increase the number of participants, the questionnaire link was also widely distributed in several social media networks including Telegram and WhatsApp. This part of the survey was performed from 4 to 25 April, 2020, during which Iran was in lockdown for the epidemic. We classified the provinces based on food security situation to food insecure (deprived), semi-secure (semi-deprived) and secure (non-deprived) [11] for further inter-provincial comparisons.

Data about the questionnaire have been comprehensively presented previously [6, 10]. Briefly, each respondent had to complete the questionnaire for his/her household. Questions were asked about socioeconomic situation (SES), income and eating habits before and during the lockdown. Also, presence of high-risk persons in the household (children under 5 years of age, pregnant or lactating women, people over 65 years and plus) and a person with the history of Covid-19 within the household were also questioned. In the nutrition section, the questions were about the change of frequency in consumption of the selected food items and the reasons for changing consumption frequency during the epidemic. In this study, only milk, yogurt and cheese were considered as dairy products.

## Ethical issues

All questionnaires were anonymous. This study was approved by the Ethics Committee of the National Nutrition and Food Technology Research Institute (IR.SBMU.NNFTRI.REC.1399.066).

### Statistical analysis

The descriptive analysis was performed to assess the distribution of socio-demographic status among respondents. Ordinal logistic regressions were fitted to examine which factors contributed to changes and decrease in selected dairy product. A 2-tailed p < 0.05 was considered significant. Two outcomes were considered to be dependent variables in regression models: 1-changes of weekly consumption frequency of milk, yogurt and cheese (more vs. no changes vs. less) 2- decrease in consumption frequency of the mentioned dairy products (little decrease vs. half vs. omitted) after the test for overall parallel assumption at 0.05 significance indicated that the overall parallel assumption has not been violated (p = 0.515 and p = 0.133).

The sex of the household’s head (male, female), living in urban/rural areas (urban, rural), household size (one to two, three to five, six and more), presence a high risk member in the household (none, under five years old, pregnant/ lactation, elder, more than one high risk member), occupation of the head (employee, freelance, retired, health worker, teacher, driver, other), educational status of the head (Master/ higher, Bachelor, Associate Degree, Diploma, High school, Hozavi (= preacher)), change in income (no changes, small decrease, half, cut), Covid-19 in family (no, yes) and food security status of the province (secure, semi secure, deprived) were the independent variables assessed. In all analyses, sampling weights were used to account for the complex sampling design and to allow inferences valid for the population. All analyses were performed using Stata version 16.0 (StataCorp LLC).

## Results

In total, 21,290 households were included in the analyses. Table 1 shows the socio-demographic characteristics of the participants. About 41% of the respondents were the household’s head, 42% were the spouse and the rest were mostly offspring. The mean (95% confidence interval (CI)) of age of households’ heads was 44.7 (44.2, 44.9) and the data indicate that 10% of the households was from rural areas.

**Table 1:** Characteristic of participants

Variable		n (%)^1^
**Sex of the head of the household**	Male Female	19,255 (89.8) 2035 (10.2)
**Urban/Rural**	Urban Rural	14,191 (73.8) 7099 (26.2)
**household size**	1–2 3–5 > 6	2883 (15.7) 16,798 (78.2) 1609 (6.1)
**High risk members in household**	None Under five years old Pregnant/ lactation Elder More than one member	11,511 (52.6) 4881 (21.8) 660 (2.8) 2110 (0.4) 2128 (8.7)
**Occupation**	Officer Freelance Retired Health workers Teacher Driver Other	3942 (20.5) 7755 (34.3) 1988 (11.7) 572 (2.7) 715 (3.1) 883 (3.9) 5435 (23.8)
**Education**	Under diploma Diploma Associate Bachelor Master/higher	8113 (33.0) 5277 (24.1) 1540 (7.3) 4022 (21.2) 2338 (14.4)

^1^ Un-weighted number, weighted percentages

**Fig. 1:**
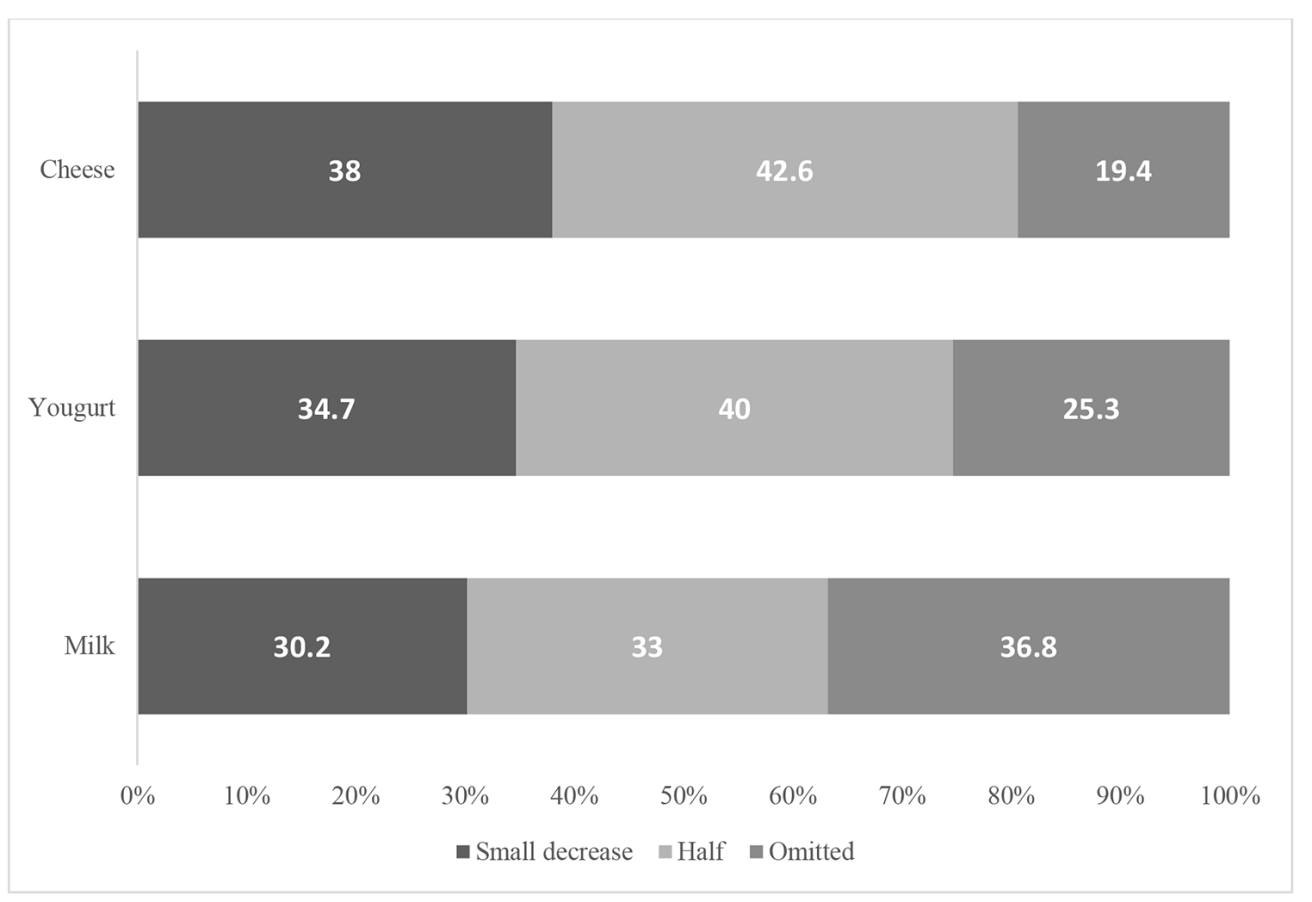
The amount of decrease in milk, yogurt and cheese consumption among those households who reported decrement in consumption frequency of these dairy products during Covid-19 epidemic lockdown

During Covid-19 epidemic lockdown, about 29%, 26% and 7% of the households had decreased their consumption frequency of milk, yogurt and cheese, respectively (Fig. 1; Table 2). The results of ordered logistic regression are presented in Table 3. The dependent variables were changes in consumption frequency of milk, yogurt and cheese following Covid-19 epidemic (increase in consumption, no changes, and decrease in consumption). The data showed that the female-headed households were 21% more likely to decrease their consumption of milk, compared with male-headed households (OR = 1.21, 95% CI: 1.05–1.4). Number of family members was not a predictor of change in the consumption frequency of milk and yogurt. However, we found that the change in income was an important predictor of changes in the consumption frequency of dairy products.

**Table 2:** Changes in consumption of milk, yogurt and cheese in household per week during Covid-19 epidemic lockdown

Variables	Changes status	n(%)^1^
**Milk**	No changes Decrease Increase	13,428 (62.5) 6293 (29.2) 1569 (8.3)
**Yogurt**	No changes Decrease Increase	14,444 (67.5) 5569(26.2) 1277 (6.3)
**Cheese**	No changes Decrease Increase	16,600 (78.2) 3873 (7.0) 817 (4.8)

^1^ Unweighted number, weighted percentages

**Table 3:** Ordered logistic regression models of changes in intake of milk, yogurt and cheese during Covid-19 epidemic lockdown

Variables	Milk		Yogurt		Cheese	
	**OR (95%CI)**	**p-value**	**OR (95%CI)**	**p-value**	**OR (95%CI)**	**p-value**
**Sex of the household head**						
Male female	- 1.21 (1.05, 1.4)	- 0.007	- 1.05 (0.91, 1.21)	- 0.494	- 1.17 (0.99, 1.38)	0.052
**Urban/Rural**						
Urban Rural	- 0.83 (0.76, 0.91)	- < 0.001	- 0.79 (0.71, 0.86)	- < 0.001	- 1.07 (0.97, 1.2)	0.157
**Family members**						
1–2 3–5 > 6	- 1.01 (0.90, 1.14) 1.12 (0.95, 1.31)	- 0.810 0.173	- 0.93 (0.82, 1.05) 0.93 (0.79, 1.11)	- 0.272 0.456	- 0.97 (0.84, 1.11) 1.02 (0.84, 1.24)	
**High risk members**						
No < 5 years old Pregnant/lactating mothers Elder More than one	- 1.12 (1.02, 1.23) 0.91 (0.71, 1.17) 1.05 (0.91, 1.20) 1.40 (1.23, 1.59)	- 0.013 0.488 0.473 < 0.001	- 1.09 (0.99, 1.19) 1.02 (0.80, 1.30) 1.12 (0.97, 1.29) 1.18 (1.03, 1.35)	- 0.078 0.828 0.102 0.012	- 1.19 (1.07, 1.34) 1.25 (0.97, 1.62) 1.16 (1.0, 1.36) 1.28 (1.1, 1.48)	- 0.002 0.082 0.049 0.001
**Occupation**						
Employee Freelance Retired Health workers Teacher Driver other	- 0.62 (0.54, 0.72) 1.03 (0.87, 1.23) 0.80 (0.62, 1.04) 1.10 (0.87, 1.40) 0.89 (0.71, 1.12) 0.84 (0.72, 0.98)	- < 0.001 0.658 0.110 0.394 0.351 0.028	- 0.58 (0.50, 0.68) 1.03 (0.86, 1.24) 0.75 (0.57, 0.97) 1.07 (0.83, 1.37) 0.72 (0.57, 0.91) 0.78 (0.67, 0.91)	- < 0.001 0.690 0.031 0.585 0.006 0.002	- 0.66 (0.56, 0.79) 1.08 (0.87, 1.32) 0.72 (0.50, 1.03) 1.33 (1.01, 1.76) 0.74 (0.57, 0.98) 0.89 (0.74, 1.06)	- < 0.001 0.458 0.080 0.041 0.036 0.198
**Change in income**						
No changes Low decrease Half Cut	- 1.36 (1.22, 1.52) 2.27 (2.03, 2.54) 5.02 (4.44, 5.67)	- < 0.001 < 0.001 < 0.001	- 1.36 (1.22, 1.52) 2.49 (2.21, 2.8) 4.9 (4.35, 5.59)	- < 0.001 < 0.001 < 0.001	- 1.37 (1.21, 1.54) 2.33 (2.02, 2.68) 5.07 (4.4, 5.83)	- < 0.001 < 0.001 < 0.001
**Covid-19 in family**						
No Yes	- 0.85 (0.66, 1.08)	- 0.205	- 1.11 (0.87, 1.40)	- 0.381	- 1.09 (0.81, 1.45)	- 0.550
**Education**						
Master./ higher Bachelor Associate Diploma High school	- 1.36 (1.16, 1.60) 1.62 (1.33, 1.99) 1.52 (1.29, 1.79) 1.67 (1.41, 1.99)	- < 0.001 < 0.001 < 0.001 < 0.001	- 1.21 (1.03, 1.42) 1.42 (1.15, 1.75) 1.46 (1.23, 1.73) 1.49 (1.24, 1.78)	- 0.018 0.001 < 0.001 < 0.001	- 1.33 (1.1, 1.16) 1.54 (1.21, 1.97) 1.64 (1.35, 1.99) 1.76 (1.43, 2.17)	< 0.001 < 0.001 < 0.001 0.003
**Security status of province**						
Secure Semi secure Deprived	- 1.11 (1.01, 1.23) 1.29 (1.15, 1.45)	- 0.043 < 0.001	- 1.08 (0.97, 1.20) 1.20 (1.07, 1.35)	- 0.143 0.002	- 1.17 (1.03, 1.33) 1.45 (1.26, 1.66)	- 0.011 < 0.001

The households residing in food insecure provinces were 29%, 20% and 45% more likely to decrease their consumption of milk, yogurt and cheese as compared with those living in the food secure provinces.

The educational status of the household’s head was also statistically associated with changes in consumption frequency of the selected dairy products after Covid-19 epidemic.

About 37%, 25.3%, 19.4% of those households who reported a decrease in consumption of dairy products had fully omitted them from their food baskets.

The ordinal regression analysis was performed to identify factors in the entire study population that were associated with amount of reduction in milk, yogurt and cheese consumption. The analysis confirmed that the assumption of parallel odds was not violate (milk, p = 0.133, yogurt, p = 0.187, cheese, p = 0.098). Therefore, results are reported for the ordered logistic models in Table 4. The analysis revealed that living in rural areas was not associated with more amount of reduction in dairy product consumption. Results indicated that people who lived in semi-secure or deprived provinces were more likely to omit their milk, yogurt and cheese intake (odds ratio (OR), 1.29, 95% CI: (1.15, 1.45), OR, 1.20, 95% CI: (1.07, 1.35), OR, 1.45, 95% CI: (1.26, 1.66) respectively). Households with cut incomes were more likely to omit their dairy products from their food baskets.

**Table 4:** Ordered logistic regression models of the determinants of reduction in milk, yogurt and cheese consumption during Covid-19 epidemic lockdown

Variables	Milk		Yogurt		Cheese	
	**OR (95%CI)**	**p-value**	**OR (95%CI)**	**p-value**	**OR (95%CI)**	**p-value**
**Sex of the household head**						
Male female	- 0.83 (0.67, 1.03)	- 0.094	- 0.80 (0.64, 1.0)	- 0.051	- 0.98 (0.74, 1.30)	- 0.929
**Urban/Rural**						
Urban Rural	- 1.1 (0.96, 1.28)	- 0.129	- 0.96 (0.83, 1.12)	- 0.656	- 1.18 (1.0, 1.40)	- 0.050
**Family members**						
1–2 3–5 > 6	- 0.80 (0.65, 1.01) 0.89 (0.67, 1.1)	- 0.052 0.399	- 0.89 (0.72, 1.10) 1.03 (0.77, 1.37)	- 0.301 0.802	- 0.74 (0.57, 0.97) 0.89 (0.65, 1.24)	- 0.029 0.520
**High risk members**						
No < 5 years old Pregnant/lactating mothers Elder More than one	- 1.06 (0.91, 1.24) 0.90 (0.62, 1.32) 0.95 (0.76, 1.19) 1.24 (1.02, 1.52)	- 0.439 0.620 0.710 0.026	- 1.19 (1.01, 1.41) 0.86 (0.57, 1.30) 0.95 (0.75, 1.20) 1.25 (1.01, 1.57)	- 0.033 0.501 0.702 0.044	- 1.29 (1.05, 1.59) 0.71 (0.45, 1.12) 0.98 (0.74, 1.28) 1.60 (1.23, 2.07)	- 0.014 0.143 0.897 < 0.001
**Occupation**						
Employee Freelance Retired Health workers Teacher Driver other	- 0.86 (0.64, 1.01) 1.38 (1.03, 1.83) 1.09 (0.75, 1.57) 1.13 (0.81, 1.57) 0.91 (0.83, 1.31) 1.05 (0.83, 1.31)	- 0.071 0.026 0.635 0.460 0.596 0.668	- 0.88 (0.68, 1.14) 1.22 (0.92, 1.63) 0.96 (0.62, 1.50) 0.95 (0.66, 1.37) 0.93 (0.66, 1.31) 1.10 (0.85, 1.42)	- 0.352 0.157 0.887 0.809 0.709 0.430	- 0.80 (0.58, 1.10) 0.84 (0.57, 1.25) 1.05 (0.62, 1.76) 0.83 (0.53, 1.31) 0.66 (0.43, 1.01) 0.86 (0.63, 1.18)	- 0.177 0.406 0.848 0.442 0.057 0.365
**Change in income**						
No changes Low decrease Half Cut	- 1.09 (0.89, 1.34) 1.54 (1.27, 1.86) 2.28 (1.87, 2.77)	- 0.371 < 0.001 < 0.001	- 0.84 (0.66, 1.06) 1.15 (0.93, 1.43) 1.82 (1.46, 2.27)	- 0.147 0.186 < 0.001	- 0.72 (0.53, 0.98) 1.08 (0.84, 1.38) 1.47 (1.15, 1.88)	- 0.037 0.523 0.002
**Covid-19 in family**						
No Yes	- 0.98 (0.73, 1.32)	- 0.941	- 1.21 (0.85, 1.72)	- 0.278	- 0.74 (0.49, 1.11)	- 0.149
**Education**						
Master./ higher Bachelor Associate Diploma High school	- 1.14 (0.88, 1.47) 1.39 (1.01, 1.91) 1.10 (0.84, 1.43) 1.29 (0.98, 1.71)	- 0.312 0.038 0.479 0.064	- 0.98 (0.75, 1.29) 1.03 (0.74, 1.42) 0.91 (0.69, 1.21) 1.07 (0.80, 1.44)	- 0.928 0.846 0.546 0.606	- 1.36 (0.94, 1.96) 1.27 (0.81, 2.0) 1.24 (0.87, 1.78) 1.23 (0.86, 1.77)	- 0.242 0.228 0.294 0.096
**Security status of province**						
Secure Semi secure Deprived	- 1.17 (1.0, 1.36) 1.18 (1.01, 1.41)	- 0.046 0.045	- 1.04 (0.88, 1.24) 0.95 (0.78, 1.15)	- 0.587 0.621	- 1.12 (0.90, 1.39) 1.17 (0.92, 1.48)	- 0.283 0.182

## Discussion

This nationwide survey was conducted for the first time to evaluate dietary changes, including dairy products consumption, following lockdown due to Covid-19 epidemic in Iran. Our data regarding the mean household size and the ratio of urban to rural households are fully in accord with those in the latest population census report in Iran [12] suggesting that our study population was representative (~ 1%) of the households in the country.

We found that some 30% of the studied household had decreased their milk and/or yogurt consumption whereas only 7% had decreased their consumption frequency of cheese. In Iran, bread and cheese are the usual constituents of a breakfast so it was not surprising that cheese consumption showed the least change. Data regarding changes of dairy consumption during pandemic are not consistent. A study from China reported that milk consumption in those subjects who returned to work during two weeks after lockdown did not change while in those who returned to work within the third week or those who stayed at home decreased significantly. Yogurt consumption did not decrease but in those who stayed at home more than three weeks during lockdown [13]. However, an analysis of consumer survey data during first Covid-19 lockdown from Denmark, Germany and Slovenia reported a minimal change in consumption of dairy products [14] and at least in one study from Italy, increased consumption of long-life milk was observed [15].

We found that female-headed, as compared with male-headed, households were significantly more likely to decrease their milk consumption. A similar study from Denmark also reported that dietary changes during lockdown period was more prominent in females than in males [16]. Notwithstanding, the root causes of dietary changes, including reduction of dairy products consumption, in Iran might be quite different including their lower labor force participation and hence more economic vulnerability and hardship [17, 18].

The finding that households residing in food insecure or semi-secure provinces were significantly more likely to decrease their dairy products consumption is of great concern. Food insecurity has adverse effects on dietary quality with consequent lower intake of nutrient-dense foods including dairy products [19]. It is believed that under conditions of food insecurity, nutrient-dense foods that may be more expensive are substituted with cheaper lower quality but usually more energy dense foods [20]. This can lead to several chronic diseases including obesity, diabetes and lower bone density [21, 22]. Actually, food insecurity and low nutritional literacy are predictors of low intake of calcium rich dairy products [23].

Two major issues are raised here. Firstly, are the observed changes in dairy products consumption in Iranian households transient or they may turn to a new dietary habit? And secondly, what will be the overall health outcomes? A very important difference in Covid-19-induced changes in dietary habits in Iran, as compared with other countries, is the concomitance of the epidemic with highly accelerated inflation in Iran [24]. Consequently, even with rather high population coverage of vaccination [25] and returning social relations and businesses almost to pre-epidemic conditions in Iran, it seems plausible that changes in dietary habits, including decreased dairy intakes, persist as a secular trend. The importance of this issue will be manifold when we consider that the pre-epidemic dairy products consumption in the country was far less the global average (~ 165 g/d per capita) [26]. This amount is also very far from the recommended intake of three servings a day [8]. The overall health outcomes of decreased dairy products consumption in Iran population can, therefore, be detrimental. Dairy products are good sources of high quality protein, fatty acids, vitamins and minerals including bioavailable calcium [27]. The beneficial effects of dairy products on bone and metabolic health have already been documented [8, 9]. Inadequate intake and, in some households, omission of dairy products can potentially bring about serious health outcomes with heavier economic burden.

Some limitations of this study are acknowledged. The questions regarding dietary intakes were qualitative with emphasis on consumption frequency and if any changes happened in the household’s usual intake. Consequently, those households with almost no milk or yogurt in their food baskets before coronavirus epidemic would report “no change” of their usual intake. Nonetheless, the noticeable proportion of the households who reported decreased or ceased consumption of milk and yogurt will well reflect the deep impact of the epidemic on their dietary habits. The agreement of responses between self-administered and interviewer-administered questionnaires was not evaluated, either. Moreover, there was a diversity in respondents but the majority of them (83%) were either the household’s head or the spouse. Further studies to track these changes over time and to evaluate their health consequences are warranted.

## Conclusion

In this nationwide cross-sectional survey, we found considerable decrement of dairy products consumption, especially milk and yogurt, in a high proportion of the studied households. Households residing in the food insecure or semi-secure provinces were more likely to be affected. Considering the accelerated inflation which adversely affects healthy foods accessibility in the country, these changes may become permanent with consequent unfavorable effects on both population health and dairy industries [28]. Early interventions including fiscal policies and subsidies for dairy products, food basket aids and supplementation are advisable to the policymakers. Though calcium supplementation may seem a very feasible and fast approach to the health decision makers, sustainability of calcium supplementation is a great challenge [29]. Meanwhile, some evidence indicates higher bioavailability of dairy products calcium, as compared with calcium supplement, for bone mineralization with a longer lasting effect on bone health [30]. Furthermore, dairy products are much more than just a “package of calcium”. They are good sources of other minerals, vitamins and high quality protein, as well [27]. Improvement of nutritional literacy of the population must also be considered as an effective strategy. It has been shown that increasing the awareness of the consumers positively affects their dairy products consumption [31].

## Data Availability

The datasets generated and/or analyzed during this study are not publicly available because of the use of data for further publications but are available from the corresponding author upon reasonable request.
